# Preparation of Testicular Cells for Immunofluorescence Analysis of Manchette in Elongating Spermatids

**DOI:** 10.21769/BioProtoc.5340

**Published:** 2025-06-20

**Authors:** Changmin Niu, Opeyemi Dhikhirullahi, Zhibing Zhang

**Affiliations:** 1 School of Nursing·School of Public Health, Yangzhou University, Yangzhou, Jiangsu, China; 2 Department of Physiology, Wayne State University School of Medicine, Detroit, MI, USA; 3 Department of Obstetrics & Gynecology, Wayne State University, Detroit, MI, USA

**Keywords:** Testicular cells, Manchette, Sperm flagella, Intra-manchette transport (IMT), Intra-flagellar transport (IFT), Immunofluorescence

## Abstract

Immunofluorescence staining is a technique that permits the visualization of components of various cell preparations. Manchette, a transient structure that is only present in elongating spermatids, is involved in intra-manchette transport (IMT) for sperm flagella formation. Sperm flagella are assembled by intra-flagellar transport (IFT). Due to the big complexes formed by IMT and IFT components, it has been challenging to visualize these components in tissue sections. This is because the proteins that make up these complexes overlap with each other. Testicular tissue is digested by a combination of DNase I and Collagenase IV enzymes and fixed by paraformaldehyde and sucrose. After permeabilization with Triton X-100, testicular cells are incubated with specific antibodies to detect the components in the manchette and developing sperm tails. This method allows for cell type–specific resolution without interference from surrounding cells like Sertoli, Leydig, or peritubular myoid cells. Additionally, isolated cells produce cleaner immunofluorescence signals compared to other methods like tissue section/whole mount, making this method the best fit for visualizing protein localization in germ cells when spatial context is not being considered. Hence, this protocol provides the detailed methodology for isolating male mice germ cells for antibody-targeted immunofluorescence assay for confocal/fluorescence microscopy.

Key features

• The protocol includes a simple method for preparing single testicular cells for immunofluorescence analysis.

• Visualization of components in the manchette and sperm flagella using specific antibody markers.

## Graphical overview


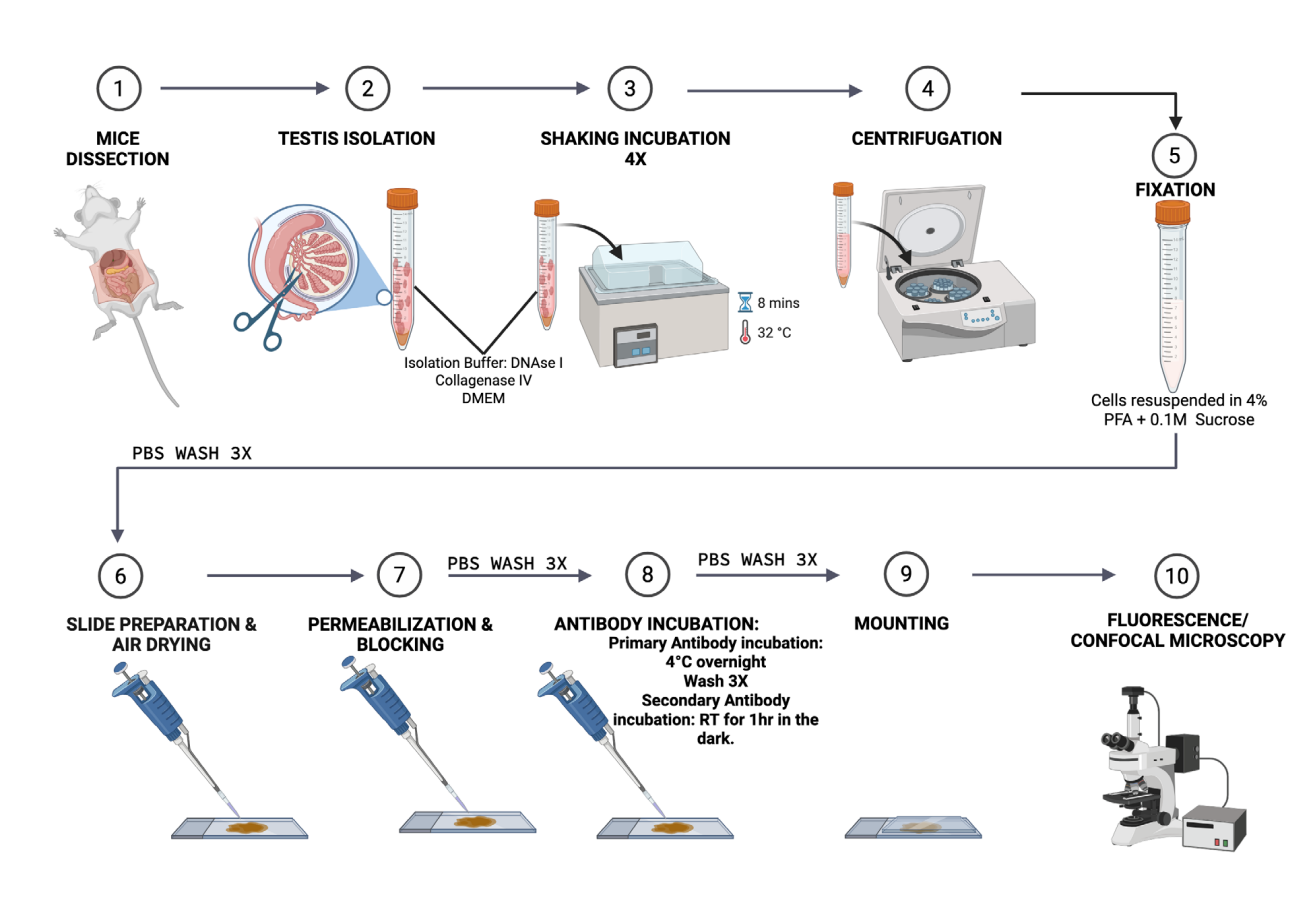


**Schematic workflow of this protocol** (figure was created using BioRender.com) https://BioRender.com/o91c792

## Background

Spermiogenesis, the final phase of spermatogenesis, entails a series of morphological transformations. During spermiogenesis, a transient structure that is only present in elongating spermatids, known as the manchette, plays a critical role in sperm flagella formation [1–2]. The manchette is enriched in microtubule/F-actin and involved in intra-manchette transport (IMT) for sperm flagella formation. This microtubule and actin-based manchette structure serves as a platform for the transport of vesicles and proteins required for the assembly of the sperm tail accessory structures, the perinuclear theca, and likely the strengthening of the head-tail attachment via IMT [3–5]. Through IMT, cargo proteins are transported to the basal bodies, where intra-flagellar transport (IFT) begins for sperm tail formation. It has been shown that disruption of the formation and function of the manchette results in a spermiogenesis defect [6–10].

The IFT complex consists of at least 22 different proteins, forming the A complex and B complex [11–14]. Several IFT components, including IFT20, IFT88, IFT140, and IFT172, have been discovered in the manchette [14–18]. Localization of these IFT components in the manchette is dependent on the normal manchette function. Thus, these IFT components might be cargo proteins of IMT, through which these IFT components are transported to the basal bodies, from where they carry sperm structural proteins for sperm flagella assembly. Abnormal sperm flagellogenesis may cause male infertility, exhibiting reduced sperm count, short and bent tails, branched flagella, disordered axoneme or microtubule doublets, and so on [7,16–18].

Immunofluorescence staining (IF) is a vital immunochemical technique that allows detection and localization of a wide variety of antigens in different types of tissues or cells by combining with specific antibodies tagged with fluorophores [19]. Although direct IF is quicker, indirect IF is more widely employed for its high sensitivity, signal amplification, and ability to detect several targets in the same sample. Indirect IF involves a two-step incubation process: 1) a primary antibody binds to the target epitope, and 2) a fluorophore-tagged secondary antibody recognizes and binds to the primary antibody. Tissue section IF allows for spatial context about germ cells, particularly early germ cells like spermatogonial cells. Based on location and association with other cells, specific cell types can be assigned to stages of spermatogenesis. Additionally, multiple cell types can be visualized in the same tube cross-section for comparative localization. However, given that the manchette and IFT components form big complexes, it is a challenge to visualize these components in the tissue sections. Thus, it is recommended to examine components in these complexes by using isolated testicular cells. While the isolated testicular cells may be used to visualize early-stage germ cells as well through cell-specific markers, we do not recommend it, particularly for spermatogonia detection, because of the loss of spatial context that might limit result interpretation.

## Materials and reagents

**Biological materials**

1. Tissue: Fresh testis isolated from C57BL/6 mice

**Reagents**

1. DNase I (Roche, catalog number: 11284932001, 100 mg)

2. Collagenase IV (Sigma-Aldrich, catalog number: C4-28-100MG)

3. DMEM (Gibco^TM^, catalog number: 11965092)

4. 10× phosphate buffer saline (PBS) (Protein Tech, catalog number: PR20014)

5. Paraformaldehyde (PFA) (Sigma-Aldrich, catalog number: 158127-500G)

6. NaOH (Millipore Sigma^TM^, catalog number: 1064621000)

7. Sucrose (Sigma-Aldrich, catalog number: S9378-500G)

8. Triton X-100 (Millipore Sigma^TM^, catalog number: TX15681)

9. Goat serum (Gibco^TM^, catalog number: PCN5000)

10. Anti-α-tubulin (Protein Tech, catalog number: 66031-1-Ig)

11. Anti-Ac-tubulin (Protein Tech, catalog number: 66200-1-Ig)

**Solutions**

1. Isolation buffer (see Recipes)

2. 1× PBS (see Recipes)

3. Fixation solution (see Recipes)

4. Permeabilization solution (see Recipes)

5. Blocking solution (see Recipes)

**Recipes**

**1. Isolation buffer**

**Table:** 

Reagent	Final concentration	Quantity or Volume
DNase I	1 μg/mL	5 μL
Collagenase IV	0.5 mg/mL	2.5 mg
DMEM	n/a	5 mL

*Note: DNase I stock solution is prepared at 1 mg/mL (10 mg of DNase I added to 10 mL of ddH_2_O) and stored at -20 °C. The stock solution needs to be thawed on ice prior to use. It is recommended that the isolation buffer be prepared fresh each time.*

**2. 1**× **PBS**

**Table:** 

Reagent	Final concentration	Quantity or Volume
10× PBS	10%	100 mL
ddH_2_O	n/a	900 mL

**3. Fixation solution**

**Table:** 

Reagent	Final concentration	Quantity or Volume
PFA	4%	4 g
1× PBS	n/a	Add to 50 mL
adjust pH to 7.7 with NaOH		
Sucrose	0.1 M	3.423 g
1× PBS	n/a	Make up to 100 mL

*Note: PFA is volatile and unstable; it is recommended to use fresh PFA each time. After preparation, it can be temporarily stored at -80 °C for one month. Before use, the frozen PFA can be thawed in a 42 °C water bath. Place on ice once thawed.*

**4. Permeabilization solution**

**Table:** 

Reagent	Final concentration	Quantity or Volume
Triton X-100	1%	10 μL
ddH_2_O	n/a	990 μL

*Note: Triton X-100 is stable and can be long-term stored at 2–8 °C under sealed and dark conditions. Triton X-100 appears slightly viscous. To aspirate Triton X-100 easily and accurately, it is recommended to cut the end of the tip.*

**5. Blocking solution**

**Table:** 

Reagent	Final concentration	Quantity or Volume
Goat serum	10%	1 mL
1× PBS	n/a	9 mL

**Laboratory supplies**

1. 15 mL Falcon tube (Falcon^TM^, catalog number: 352095)

2. Pap pen (Scytek Laboratories, catalog number: LP0002)

3. Slide (Thomas Scientific, catalog number: 1158B91)

4. Kim Wipe (Kimberly-Clark, USA)

5. Mounting media (VECTASHIELD^®^ Antifade Mounting Medium with DAPI, catalog number: H-1200-10)

6. Cover slip (epredia, catalog number: 102222)

7. Nail polish (Superdry^®^)

## Equipment

1. Transfer pipette (Fisherbrand, catalog number: 13-711-9AM)

2. Centrifuge (Eppendorf^TM^, model: 5810R)

3. Laser scanning confocal microscopy (Zeiss, model: LSM 700)

## Software and datasets

1. ImageJ (free, https://imagej.net/software/fiji/downloads, Fiji version, 2024)

2. GraphPad Prism 8.0.2

## Procedure

All procedures are carried out at room temperature unless otherwise specified.

**A. Isolation of mouse testes**

**Figure 1.**
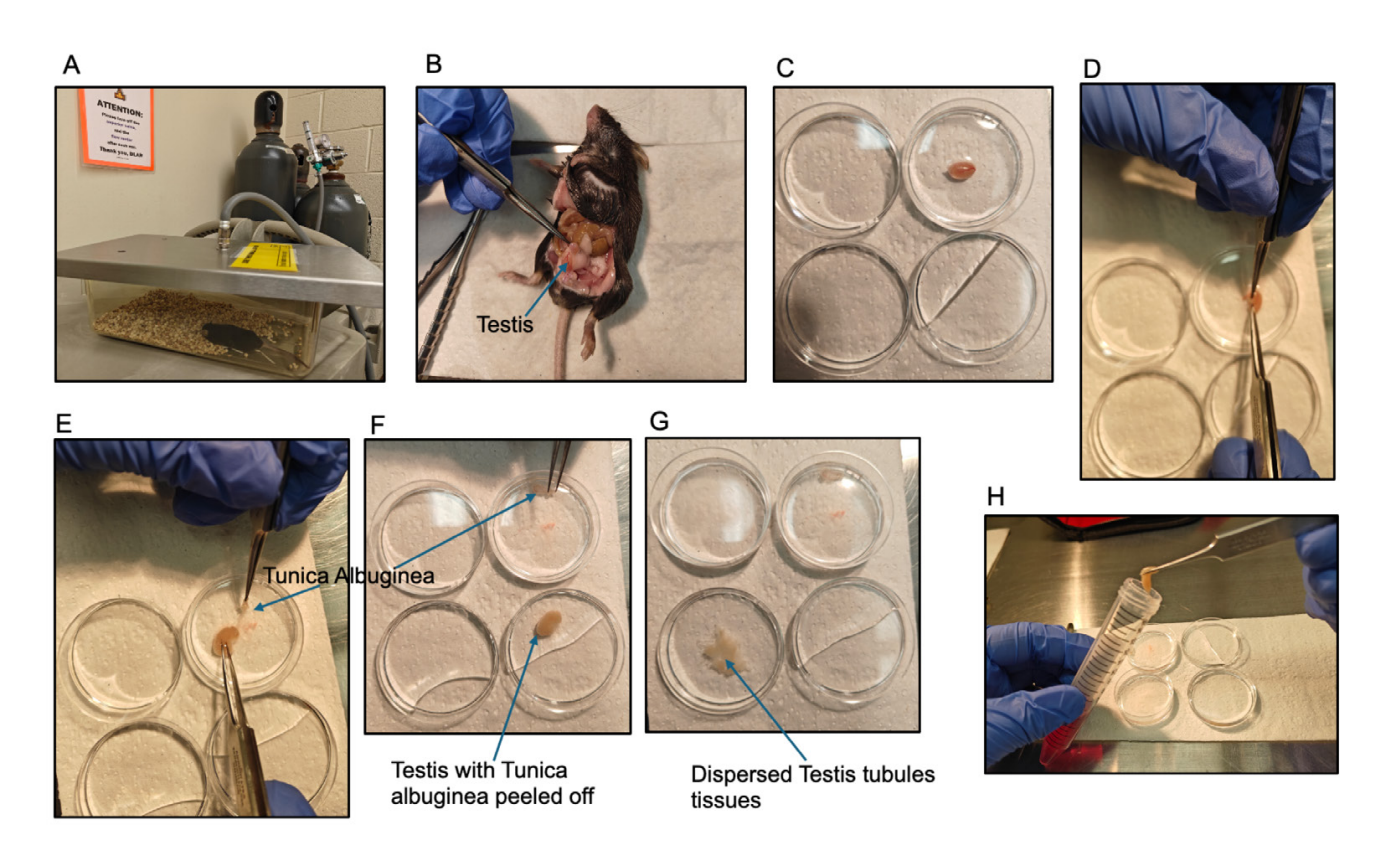
Stepwise isolation of mouse testis for germ cell retrieval. (A) Male mice anesthetization in an adequate chamber before cervical dislocation. (B) Abdominal incision to reveal the testes. (C) Isolated testis in cold PBS. (D–F) Removal of the tunica albuginea from the testis before digestion. (G–H) Testicular tissue cut up into pieces to disperse the seminiferous tubules before transfer into isolation buffer.

1. Set up your dissection area in a clean and well-lit animal treatment room/workspace.

2. Put male mice in a suitable chamber/container for anesthetization using CO_2_ as shown in Figure 1A.

3. Ensure the mouse is completely unconscious before taking it out of the container/chamber. This can be confirmed by tapping the mouse directly or shaking the container/chamber gently to monitor movement. Lack of movement is an indication of unconsciousness. Sacrifice mice by cervical dislocation.

4. Place mice with their abdominal side facing upward. Use scissors to make an incision to expose the lower abdomen. Pull out the testes and remove the fat and epididymis attached to them.

*Note: One testis is usually more than enough to get germ cells per experiment.*

5. Put the testes in cold PBS to wash. Then, transfer to fresh cold PBS to remove the membrane (tunica albuginea) surrounding it, as shown in Figure 1D–G.

6. Cut testes into tiny pieces. Then, transfer into a 15 mL Falcon tube containing 5 mL of isolation buffer to digest.

**B. Shaking incubation**

1. Place the tube in a 32 °C water bath and shake gently (50 rpm) for 8 min. Use a transfer pipette to gently blow tissues off the bottom of the tube every 3 min.

2. Repeat step B1 three more times.

**Figure 2.**
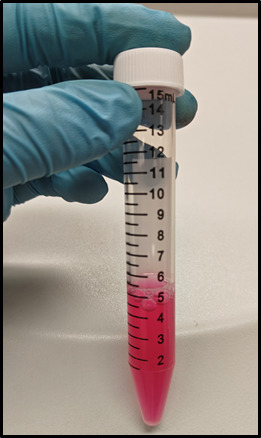
Representative image showing well-digested testicular tissues following 32 min of shaking incubation

*Note: Total time: 4 × 8 min =32 min. By now, the tissues should be completely dissociated, as shown in Figure 2.*

**C. Centrifugation**

1. While waiting, cool the Eppendorf^TM^ 5810R to 4 °C.

2. After four repeats of shaking incubation, place the tube into the centrifuge and spin down at 200× *g* for 5 min at 4 °C.

**D. Discarding supernatant**

1. Discard supernatant.

2. Add 5 mL of 1× PBS and gently suspend the cells with the transfer pipette.

3. Centrifuge at 200× *g* for 5 min at 4 °C.

**E. Fixation**

1. Add 5 mL of fixation solution.

2. Gently suspend the cells with the transfer pipette and shake at room temperature for 15 min on a platform shaker (50 rpm).

*Note: PFA is toxic and should be handled according to standard safe use practices. Fixation waste should be collected in designated containers adequate for chemicals for hazardous waste cleanup in accordance with institutional environmental and health safety guidelines.*

**F. Examination**

1. Centrifuge the cells at 200× *g* for 5 min at 4 °C.

2. Discard the supernatant and wash three times with 1× PBS.

3. Resuspend the cells with PBS at 1 × 10^5^/mL (1–2 × 10^6^/mL, sperm cells).

4. Add 50 μL of cell suspension to 500 μL of fresh PBS.

5. Aliquot ~10 μL of cell suspension into a Petri dish and examine the cells under a microscope.

**Figure 3.**
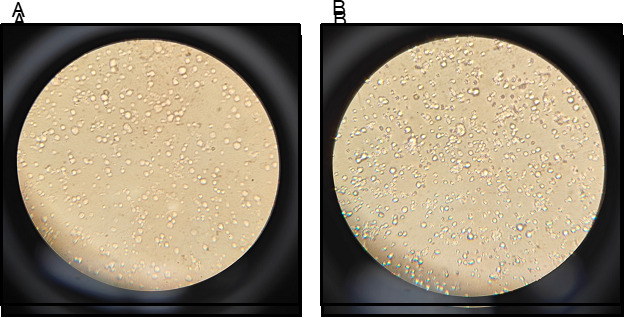
Microscope view of cell suspension on a slide for density analysis. (A) Representative image showing an adequately diluted germ cell suspension for analysis. (B) Representative image of a slide with too much cell density for analysis.

*Note: Ensure the cells are adequately diluted; they should be separated and individualized as in Figure 3A.*

**G. Preparation of slides**

1. If density is appropriate, use a PAP pen to draw a square on a positively charged slide.

2. Plate 50 μL of the prepared cell suspension from F3 onto each square.

3. Gently smear to fill the entire square and leave horizontally.

*Note: A typical experiment should contain the experimental group, a negative control, and a positive control. The number of slides depends on the type and number of antibodies that are ready to be used.*

**H. Air drying**

1. Air dry the cell suspension for 45 min to 1 h.

**Figure 4.**
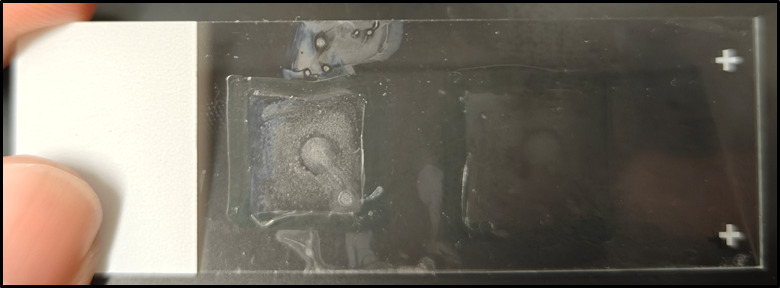
Representative image of a cell suspension that is too dry on the slide

*Note: Do not let the cell suspension dry completely, as shown in Figure 4. This is to prevent nonspecific signals. Also, if the samples are too dry, binding of antibodies to the antigen will be impaired.*

2. Insufficient drying before performing the next step might lead to cell loss.

3. Optimal dryness is achieved when the sample area is still wet, but there is no extra layer of liquid on the slide.

**I. Permeabilization**

**Figure 5.**
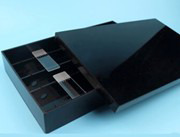
Image of a wet box with slides in it. Put slides in the wet box during incubation to prevent the slides from drying out.

1. Place sliders in a wet box (Figure 5). Permeabilize the cells with 50 μL of Triton X-100 (1%) at 37 °C for 5 min.

*Note: The wet box, also known as a humidity chamber, is a container or device designed to maintain a specific level of humidity within a confined space. Inside the box, there are mechanisms to control and regulate humidity. This can be achieved through the use of water-retaining substances, such as wet sponges or water-filled reservoirs, which release moisture into the air as it evaporates. The wet box is then placed in an incubator at 37 °C.*

**J. Blocking**

1. Wash the cells gently three times with 1× PBS.

2. Add 50 μL of blocking solution to each square and incubate the cells at 37 °C for 30 min in the wet box.

**K. Antibody incubation**

1. Gently remove the blocking solution using a 100 μL pipette, add the indicated primary antibodies to the cells, and incubate overnight at 4 °C in a wet box.

2. Gently wash the cells three times with 1× PBS.

3. Incubate the indicated secondary antibodies in a wet box at room temperature for 1 h.

4. Gently wash the cells three times with 1× PBS for a total of 15 min. Gently use a Kimwipe to dry clean the surface of the slide.

*Notes:*

*1. Washing is very important and may need to be prolonged. Severe washing will make the cells fall off. Washing may be achieved by using a pipette to load PBS onto the slides at the edge of the square, followed by gentle aspiration from the edge to minimize cell loss during washing.*

*2. Keep in a wet box to prevent slides from drying, and then in a drawer to avoid light and drying. Fluorescent substances tend to experience a gradual decrease in fluorescence intensity when exposed to light, especially intense light. The fluorescent markers used in immunofluorescence experiments, such as FITC (fluorescein isothiocyanate) and TRITC (tetramethylrhodamine isothiocyanate), undergo photochemical reactions under continuous excitation light, which leads to the destruction of their molecular structures and the subsequent loss of their fluorescent properties. If light is not avoided during the experiment, the premature quenching of the fluorescent markers will affect the observation and detection of fluorescent signals, reducing the sensitivity and accuracy of the experiment.*

**L. Mounting**

1. Add mounting media (VECTASHIELD® Antifade Mounting Medium with DAPI).

2. Slowly cover with a coverslip and seal with nail polish.

*Notes:*

*1. Keep in the dark to avoid light.*

*2. DAPI is a benzimidazole derivative. It has a strong affinity for double-stranded DNA due to its ability to intercalate between the base pairs of DNA. It specifically binds to DNA, enabling clear visualization of the cell nucleus and other DNA-rich regions in cells. This high specificity makes it an excellent tool for distinguishing and analyzing the nuclear morphology and distribution of cells.*

*3. Ensure even distribution of DAPI and avoid introducing bubbles during coverage. This can be achieved by adding a drop of DAPI to the center of each sample square, then gently positioning the coverslip to the edge, and gradually tilting down to cover the slide. Then, press down gently for an even spread and the removal of excess DAPI to the side. Remove excess DAPI gently with wipes while ensuring the coverslip is not displaced.*

**M. Imaging**

Observe and capture images using a fluorescence microscope or a confocal laser scanning microscope.

## Data analysis

If needed, ImageJ (free, https://imagej.net/software/fiji/downloads , Fiji version, 2024) can be used to analyze fluorescence signal strength, area, etc., and GraphPad Prism can be used to map.

## Validation of protocol

This protocol or parts of it has been used and validated in the following research article(s):

- Yap et al. [20]. DNALI1 interacts with the MEIG1/PACRG complex within the manchette and is required for proper sperm flagellum assembly in mice. *eLife*. (Figures 4B and 8).

- Yap et al [18]. MEIG1 determines the manchette localization of IFT20 and IFT88, two intraflagellar transport components in male germ cells. *Dev Biol.* (Figure 1A, B)

- Huang et al. [21]. Autophagy core protein ATG5 is required for elongating spermatid development, sperm individualization and normal fertility in male mice. *Autophagy.* (Figure 1B).
- Zhang et al. [12]. Intraflagellar transport protein IFT20 is essential for male fertility and spermiogenesis in mice. *Mol Biol Cell.* (Figure 5B, C; Figure 6B).
- Li et al. [22]. A MEIG1/PACRG complex in the manchette is essential for building the sperm flagella. *Development.* (Figure 2C, D).

## General notes and troubleshooting

**General notes**

1. All mouse studies need to be approved by the Institutional Animal Care and Use Program Advisory Committee.

2. Testicular cells should be separated and individualized to obtain accurate staining signals.

3. Spermatogenesis begins with spermatogonial stem cells, which gradually form sperm through a complex differentiation process, including spermatogonial cells, round sperm cells, and finally elongated sperm cells. Spermatogenesis is a dynamic process; the number of spermatogenic cells in different stages of development is constantly changing, so the specific number and proportion of elongated spermatogenic cells in testis are difficult to accurately define and are generally identified by cell morphological characteristics.

4. Different marker antibodies are recommended to specifically identify cell types and structures.

**Troubleshooting**

Problem 1. Inadequate testicular cell segregation.

Cause: The testicular tissue is not fully digested.

Solution: It is recommended to prepare isolation buffer fresh each time. Ensure optimal time (32 mins) for shaking incubation.

Problem 2. Weak or no staining signal.

Causes: (1) Poor antibody specificity. (2) The fluorescence signal is quenched. (3) Low antibody concentration.

Solutions: (1) Select antibodies that are well-documented in the literature. Antibody specificity needs to be verified before the experiment. (2) Avoid light from the incubation of the second antibody at the end of the experiment. (3) According to the instructions, select the appropriate antibody concentration.

Problem 3. High background/nonspecific staining.

Causes: (1) Dried out samples. (2) Insufficient blocking.

Solutions: (1) Avoid over-drying slides. (2) Use the appropriate blocking solution for the required time.

Problem 4. Cell loss from the slide.

Causes: (1) Harsh washing. (2) Insufficient drying.

Solutions: (1) Avoid aggressive pipetting or shaking during washes. (2) Use charged or coated slides. (3) Ensure optimal dryness before permeabilization.
